# Suboptimal health: a potential preventive instrument for non-communicable disease control and management

**DOI:** 10.1186/1479-5876-10-S2-A45

**Published:** 2012-10-17

**Authors:** Wei Wang

**Affiliations:** 1 School of Medical Science, Edith Cowan University, Perth, Australia; 2 Graduate School, Chinese Academy of Sciences, Beijing, China; 3 School of Public Health and Family Medicine, Capital Medical University, Beijing, China

## 

Suboptimal health status (SHS) is characterized by ambiguous health complaints, general weakness, and lack of vitality, and it has become a new public health challenge in China [1,2]. SHS is believed to be a subclinical, reversible stage of chronic disease. As studies of intervention and prognosis for SHS are expected to become increasingly important, a reliable and valid instrument for its assessment is essential. A questionnaire for measuring SHS in urban Chinese was developed based on focus group discussions and a literature review [1]. Questionnaire validity and reliability were evaluated in a small pilot study and then in a cross-sectional study of 3000 individuals [2]. The analyses included tests for reliability and internal consistency, exploratory and confirmatory factor analysis, and tests for discriminative ability and convergent validity. The final questionnaire incorporated 25 items on SHS (SHSQ-25), and encompassed 5 subscales: fatigue, cardiovascular system, digestive tract, immune system, and mental status [1,2]. The SHSQ-25 has proved to be a reliable and valid instrument for measuring sub-health status in urban Chinese [2]. The progress of a combined genomics and glycomics study for screening biomarkers and exploring SHS as a preventive tool for non-communicable disease control and management will be presented [3].
